# Implementation of cascade logic gates and majority logic gate on a simple and universal molecular platform

**DOI:** 10.1038/s41598-017-14416-7

**Published:** 2017-10-25

**Authors:** Jinting Gao, Yaqing Liu, Xiaodong Lin, Jiankang Deng, Jinjin Yin, Shuo Wang

**Affiliations:** Key Laboratory of Food Nutrition and Safety (Ministry of Education of China), College of Food Engineering and Biotechnology, Tianjin University of Science and Technology, Tianjin Economic and Technological Development Area, the 13th Avenue, No. 29, Tianjin, 300457 China

## Abstract

Wiring a series of simple logic gates to process complex data is significantly important and a large challenge for untraditional molecular computing systems. The programmable property of DNA endows its powerful application in molecular computing. In our investigation, it was found that DNA exhibits excellent peroxidase-like activity in a colorimetric system of TMB/H_2_O_2_/Hemin (TMB, 3,3′, 5,5′-Tetramethylbenzidine) in the presence of K^+^ and Cu^2+^, which is significantly inhibited by the addition of an antioxidant. According to the modulated catalytic activity of this DNA-based catalyst, three cascade logic gates including AND-OR-INH (INHIBIT), AND-INH and OR-INH were successfully constructed. Interestingly, by only modulating the concentration of Cu^2+^, a majority logic gate with a single-vote veto function was realized following the same threshold value as that of the cascade logic gates. The strategy is quite straightforward and versatile and provides an instructive method for constructing multiple logic gates on a simple platform to implement complex molecular computing.

## Introduction

As a scientifically interesting topic, untraditional molecular computing has drawn increasing attention across extensive research fields from solving non-deterministic problems to building logic gates^1–5^. Though significant achievements have been obtained, molecular computing power is greatly restricted due to limitations on constructing cascade logic gates and integrating multiple logic gates within a simple universal platform^6–9^. In electronics, cascade logic gates can be easily made by physically connecting gates with a conductive wire to transfer the output of an up-stream logic gate as the input of a down-stream logic gate^10^. Together with incremental, cascading levels of logic gates, computing power can be improved for sophisticated data and information processing^11^. In molecular systems, however, it is a great challenge to connect logic gates with conductive wire or molecules. A recently developed toe-hold-based DNA displacement strategy has been confirmed to be an intriguing way to build multi-level cascade logic gates^12^. By combining G-quadruplex split strategy, cascade logic gates of the AND-NOT and AND-OR-YES types have been constructed with fluorescence as readout signal, which presents a potential application for logic-controlled drug release. However, waste is usually accumulated in the system. Enzyme catalysis of small molecules provides some advantages, but is limited for further development because only a few enzyme-based systems are available for the construction of concatenated logic gates^13,14^. An alternative approach is to develop a molecular system or nanomaterial that responds differently to external stimuli to implement multiple logic functions^11,15–19^. Though pioneer investigations have open diverse way for integrating multiple logic gates at the molecular scale, investigations are still at a very early stage.

DNA has been confirmed to be an excellent building block for constructing molecular logic gates to address various problems of molecular computing due to its intrinsic merits, such as easy synthesis, low cost and well-ordered and predictable structure^20^. Up to date, various DNA-based logic gates have been successfully developed, including a whole set of basic logic gates, as well as advanced logic circuits^21–25^. However, DNA-based cascade logic gates have rarely been reported. It is interesting that the catalytic activity of DNA-based catalysts is related to its sequence as well as configuration, which can be modulated by other DNA strands, proteins, metal ions and small molecules^26,27^. This provides significant opportunity for developing distinct logic gates. Herein, we propose the use of guanine-rich DNA (G-DNA) combined with metal ions and an antioxidant to modulate DNA-based catalytic activity on a colorimetric system of TMB (3,3′,5,5′-tetramethylbenzidine) for a multi-level cascade logic operation including AND-OR-INH (INHIBIT), AND-INH and OR-INH. Furthermore, a majority logic gate with a single-vote veto function was realized by modulating the concentration of metal ions.

## Results and Discussion

### Operation mechanism of the developed logic gates

**Figure 1:**
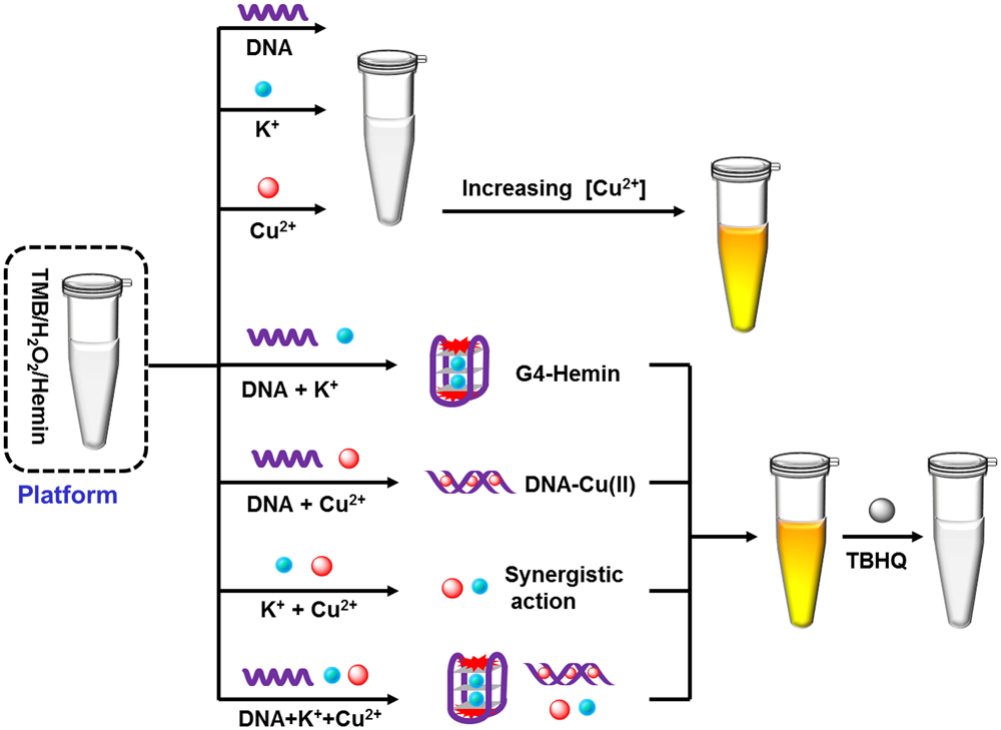
Operation principles for the developed cascade logic gates and majority logic gate with single-vote veto. G-DNA and K^+^ has low catalytic activity and cannot cause obvious color change of the platform. The peroxidase-like activity of Cu^2+^ is related to the concentration of Cu^2+^, which is enhanced with increasing the concentration. The combinations of any two or three of G-DNA, K^+^ and Cu^2+^ exhibits excellent catalytic activity, transferring the solution color into yellow. Differently, antioxidant of TBHQ strongly inhibits the colorimetric reaction.

As an ideal peroxidase substrate, TMB is usually used as signal indicator because it can sensitively reflect structural changes through colour changes in the presence of H_2_O_2_^28–30^. To implement multiple logic gate functions, here, hemin was integrated into the colorimetric system, TMB/H_2_O_2_/Hemin, as the initial logic platform. As illustrated in Fig. 1, G-DNA and K^+^ alone presented low catalytic activity and did not cause an obvious colour change. Cu^2+^ was found to exhibit peroxidase-like activity in the colorimetric system, which was enhanced with increasing concentrations. Combinations of any two or three of G-DNA, K^+^ and Cu^2+^ showed peroxidase-like activity, changing the colour of the solution to yellow. By contrast, the antioxidant TBHQ (tertiary butyl hydroquinone) strongly inhibited the colorimetric reaction. According to this tunable catalytic activity, a series of cascade logic gates and a majority logic gate with a single-vote veto were conceptually mimicked for the first time with the TMB/H_2_O_2_/Hemin system as the logic platform. The developed logic system was able to implement a biosensor function for high-sensitivity detection of antioxidants. The experimental conditions were optimized according to the requirements of the majority logic gate and cascade logic gate (See Fig. S1 in the supporting information (SI)).

### Construction of an AND-OR-INH cascaded logic gate

**Figure 2:**
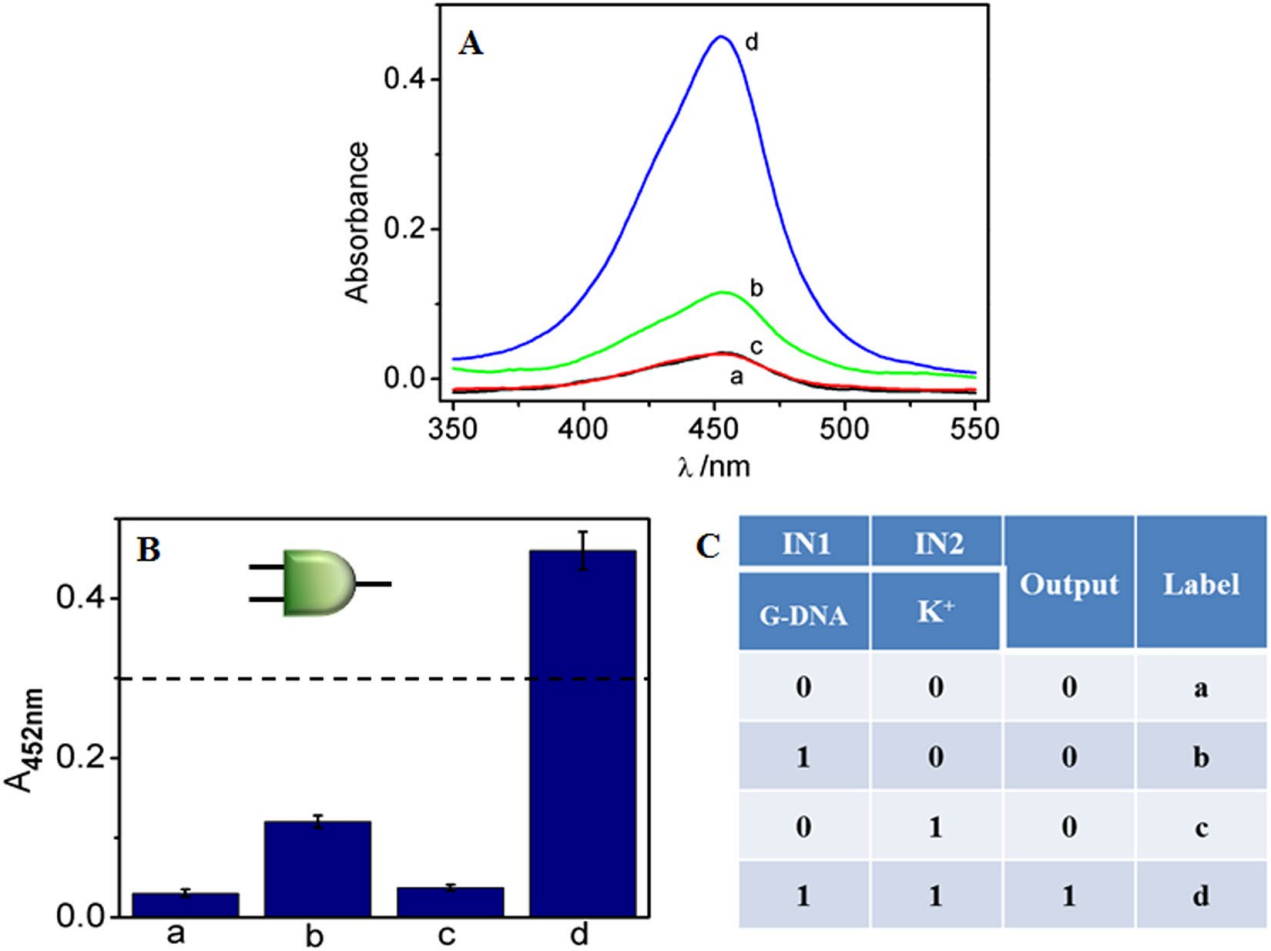
(**A**) UV-vis absorption spectra of the logic gate system in the absence of any input (a) and in the presence of IN1 (b), IN2 (**c**) and IN1 + IN2 (d). (**B**) The absorption intensity of the logic gate system at 452 nm (A_452nm_) against various input combinations. (**C**) The truth table of AND logic gate. Here, IN1 and IN2 are G-DNA (400 nM) and K^+^ (20 mM), respectively. The error bar (mean ± SD) is obtained according to three independent experimental results.

Complex computational processes can be effectively conducted via cascaded operation of multiple simple logic gates^10^. Here, the cascade logic gate starts from an AND logic gate, with G-DNA and K^+^ as the two inputs, IN1 and IN2. As illustrated in Fig. 2A, a low absorption response is monitored in the absence of any input (a) and in the presence of an individual input of G-DNA (b) or K^+^ (c). In the presence of both G-DNA and K^+^, a K^+^-stabilized G-quadruplex (G4) with a parallel configuration is formed^31^, which was confirmed by the circular dichroism experimental results (See Fig. S2 in SI). Due to the specific affinity with hemin^32^, a G4-Hemin DNAzyme is then generated, which has excellent peroxidase-like activity^33^, resulting in a high absorption response of TMB, Fig. 2A(d). To perform the AND logic gate function, the absorption intensity of TMB at 452 nm (A_452nm_) is defined as the output signal and plotted against the input combinations (Fig. 2B). The input is defined as “1” or “0” corresponding to the presence or absence of inputs, respectively. The output is defined as “1” or “0” if A_452nm_ is higher or lower than threshold value of 0.30. This definition is available for all constructed logic gates in our investigation. The corresponding logic circuit scheme and truth table are shown in the insets of Fig. 2B and Fig. 2C, respectively. The system has a high output signal only when the two inputs are both present, fulfilling the function of the AND logic gate.

**Figure 3:**
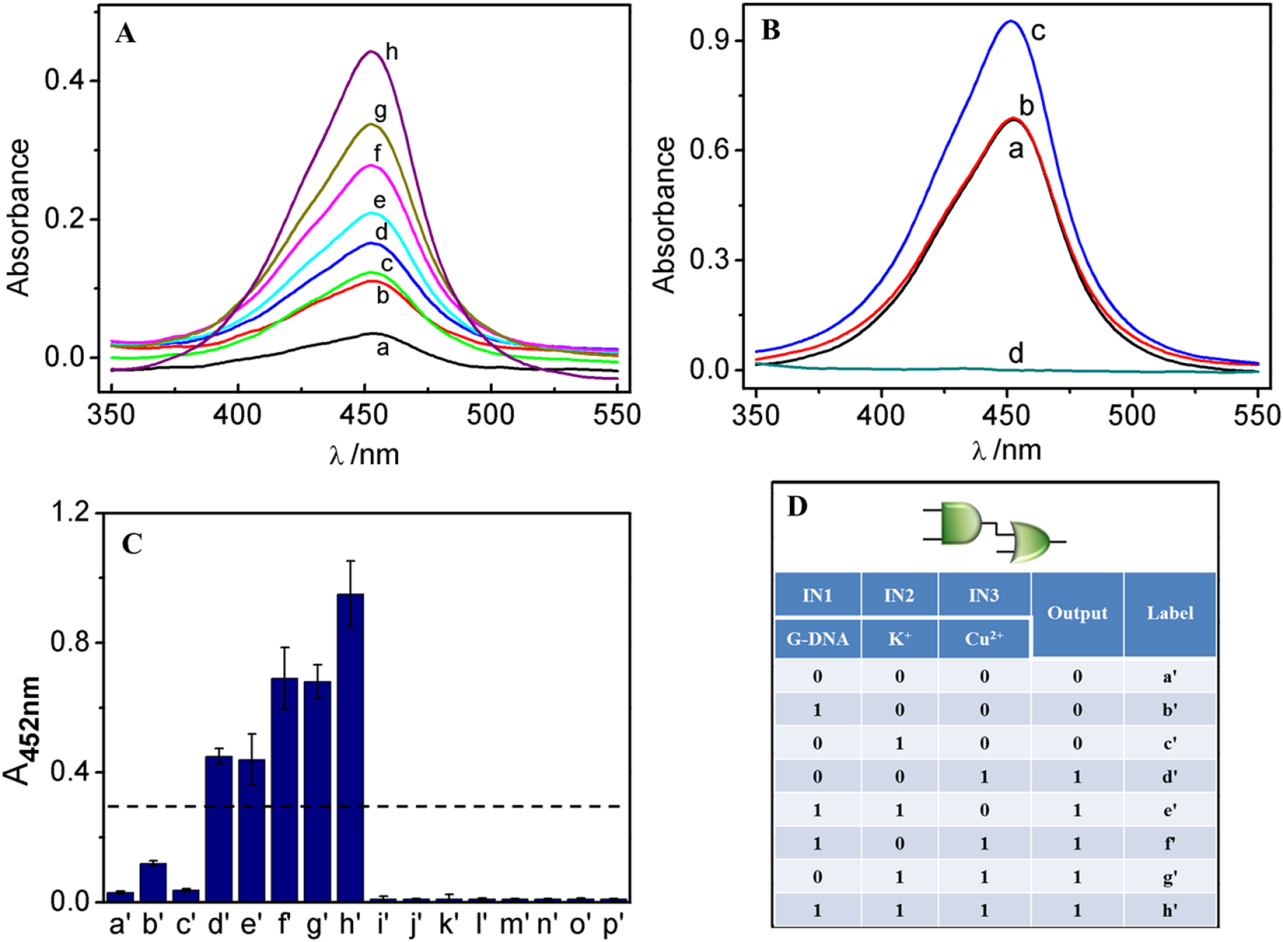
UV-vis absorption spectra of the colorimetric system against Cu^2+^ with concentrations: 0, 1, 5, 10, 20, 25, 30, 40 μM from *a* to *h* (**A**) and against input combinations: (IN1 + IN3, *a*), (IN2 + IN3, *b*), (IN1 + IN2 + IN3, *c*) and (IN1 + IN2 + IN3 + TBHQ, *d*) (**B**). Column bar of A_452nm_ against input combinations (**C**): a’) absence of the inputs, b’) IN1, c’) IN2, d’) IN1 + IN2, e’) IN3, f’) IN1 + IN3, g’) IN2 + IN3, h’) IN1 + IN2 + IN3, i’) IN4, j’) IN1 + IN4, k’) IN2 + IN4, l’) IN1 + IN2 + IN4, m’) IN3 + IN4, n’) IN1 + IN3 + IN4, o’) IN2 + IN3 + IN4, p’) IN1 + IN2 + IN3 + IN4. Here, the IN1, IN2, IN3 and IN4 are G-DNA (400 nM), K^+^ (20 mM), Cu^2+^ (40 μM) and TBHQ (10 μM), respectively. (**D**) Truth table of AND-OR logic circuit. The error bar (mean ± SD) is obtained according to three independent experimental results.

To construct the second level OR logic gate, Cu^2+^ is introduced to implement the AND-OR logic function. It was found that Cu^2+^ exhibits peroxidase-like activity on the colorimetric reaction of TMB/H_2_O_2_/Hemin, which linearly increases with increasing concentration of Cu^2+^, Fig. 3A. The possible reason for this might be that Cu^2+^ may act as Fenton-like reagent^34^ and interact with TMB in the presence of H_2_O_2_, resulting in a coloured product. To perform the AND-OR logic function, Cu^2+^, at a concentration of 40 μM, was used as the third input (IN3), resulting in a high output “1”, Fig. 3A(h). In the presence of both G-DNA (IN1) and Cu^2+^ (IN3), the peroxidase-like activity in the colorimetric system was significantly enhanced, Fig. 3B(a), which was ascribed to the synergistic action of DNA and Cu^2+^. In this case, no obvious configuration change of DNA was seen in the CD results (See Fig. S1 in supporting information (SI)). Transition metal ions such as Cu^2+^ favour Lewis acid-based interactions with electron-rich oxygen and nitrogen atoms in the DNA^21^. The coordination of Cu^2+^ with DNA produces DNA-Cu (II) complexes exhibiting excellent DNAzyme activity and resulting in a coloured product from TMB^35^. In the presence of both K^+^ (IN2) and Cu^2+^ (IN3), K^+^ would act as a promoter to promote the catalytic activity of the Cu (II)-based catalyst^36^, leading to an enhanced absorption response (Fig. 3B(b)). The coexistence of G-DNA, K^+^ and Cu^2+^ results in an even higher output signal due to the synergistic effect of the three inputs (Fig. 3B(c)). With the output of the first-level AND gate (Fig. 2) and Cu^2+^ (40 μM) as inputs of the second-level logic gate, A_452nm_ was plotted with respect to various input combinations, producing a corresponding column bar with the logic circuit scheme (Fig. 3C, from a’ to h’) and truth table (Fig. 3D). The results reveal proper implementation of an AND-OR cascade logic gate that transfers the outputs of the AND gate as inputs them into the downstream OR gate.

**Figure 4:**
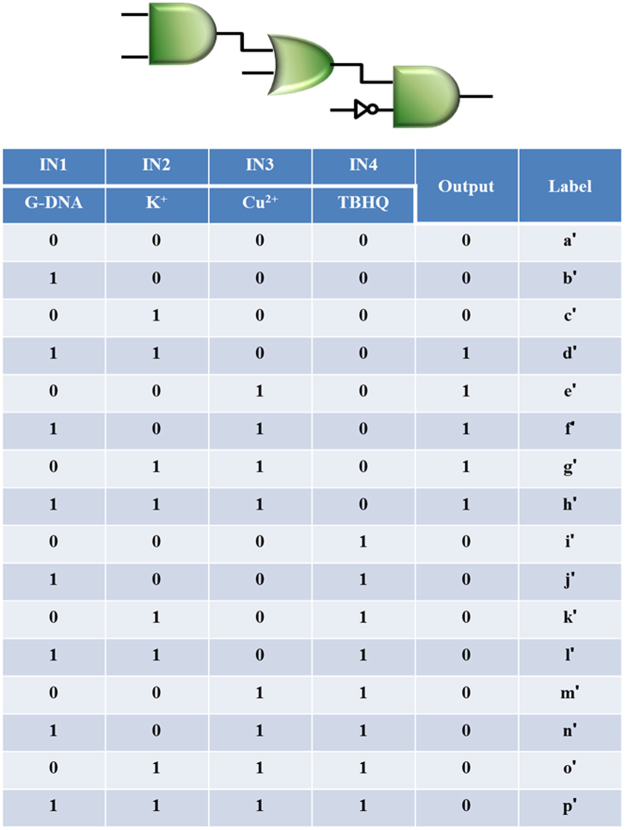
Truth table of the cascade AND-OR-INH logic gate with corresponding logic circuit.

The increment of a cascade level logic gate can improve computing power for complex data processing^11^. Here, we continued to construct a multi-level cascade logic gate by integrating the AND-OR gate with an INH logic gate. As demonstrated above, the first and second-level logic gates were implemented based on the catalytic activity of the colorimetric system. To construct the third-level logic gate, the antioxidant TBHQ was introduced as the fourth input (IN4), which can inhibit the initiation or propagation of oxidizing chain reactions by adsorbing and neutralizing free radicals, quenching singlet and triplet oxygen or decomposing peroxidase^37^. As expected, the peroxidase-like activity under the synergistic action of G-DNA, K^+^ and Cu^2+^ was significantly inhibited by TBHQ, Fig. 3B(d). A similar low absorption response was observed when TBHQ was added with any input combination. With the output of AND-OR logic gate described above and TBHQ as the input of the third-level logic gate, A_452nm_ was plotted against input combinations, Fig. 3C (from a’ to p’), generating corresponding truth table shown in Fig. 4. The logic system performs a final INH logic gate function. Thus, the full function of the multi-level logic gate corresponds to an AND-OR-INH cascade logic operation. It transfers the output of the first-level AND gate as the input of the second-level OR gate, and then downstream to the third-level INH gate to accomplish gate-to-gate communication. This logic system has the advantage that the operation is quite simple and straightforward, eliminating waste accumulation. Meanwhile, the operations of the logic gates share a common threshold value and can be implemented on a universal platform, which would be an advantage for the integration of molecular devices. With increasing computational complexity, a molecular platform capable of performing multiple logic operations is required^38^. According to the results above described, AND-INH and OR-INH cascade logic gates also can be implemented to meet the requirements of molecular computing (See Figs S3 and S4 in SI). The logic gate system also presents promise as a biosensor, such as for high sensitivity detection of antioxidants, which are important for human health and food safety (See Fig. S5 in SI).

### Construction of a majority logic gate

**Figure 5:**
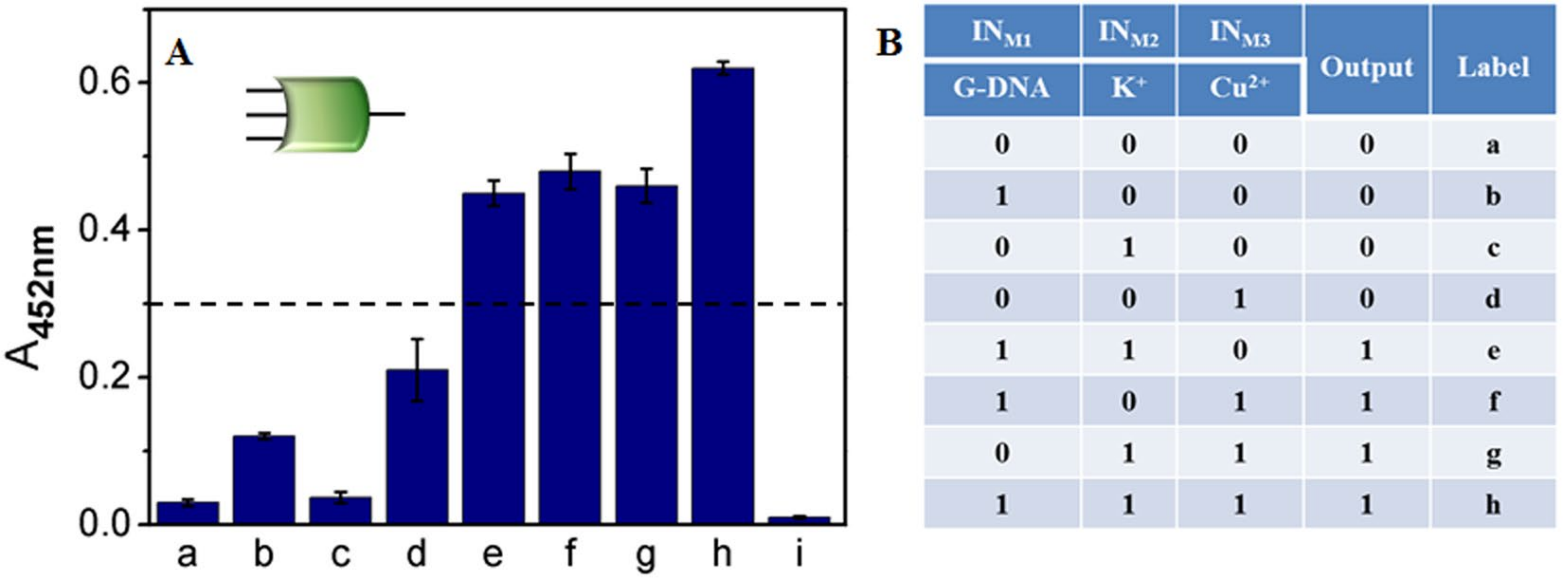
(**A**) Column bar of A_452nm_ against input combinations with logic circuit scheme. (**B**) The corresponding truth table of the majority logic gate. Here, the IN_M1_, IN_M2_ and IN_M3_ are G-DNA (400 nM), K^+^ (20 mM) and Cu^2+^ (20 μM), respectively. The error bar (mean ± SD) is obtained according to three independent experimental results.

Notably, the peroxidase-like activity of Cu^2+^ in the colorimetric system is concentration-related, which can be further modulated by combining G-DNA or K^+^ (See Fig. S6 in SI). This provides a flexible way for constructing multiple distinct logic gates. According to the experimental results, a three-input majority logic gate was also realized on the same logic platform with TMB/H_2_O_2_/Hemin. A majority logic gate reports a *TRUE* output “1” when more than half of the inputs are present. Otherwise, a *FALSE* output “0” is reported^5^. This intrinsic feature imparts a majority logic gate voting-like function and the possibility of the system being used in fault-tolerant computing and construction of more complex logic circuits^33,39^. Only a few molecular majority logic gates have been successfully constructed with elaborate design^5,33,40–42^. Here, we described the construction of a three-input majority logic gate with G-DNA and K^+^ as IN_M1_ and IN_M2_, respectively, and Cu^2+^ with concentration of 20 μM as IN_M3_ (distinct from IN3 used in the cascade logic gate). As illustrated in Fig. 5A, the logic system reports low output signals in the form of A_452nm_ in the absence (**a**) and presence of single input of IN_M1_ (**b**), IN_M2_ (**c**), or IN_M3_ (**d**). High output signals are monitored in the presence of any two inputs, (IN_M1_ + IN_M2_, e), (IN_M1_ + IN_M3_, f) or (IN_M2_ + IN_M3_, g), and all the three inputs, (IN_M1_ + IN_M2_ + IN_M3_, h). Based on the corresponding truth table, Fig. 5B, the logic system makes a *TRUE* decision only if more than half of the inputs are introduced, meeting the requirements of majority logic gate function.

For the majority logic gate, each input acting as a voter has an equal input on decision making. In some cases, however, some members may have priority over the others to vote down the proposal if they have a single-vote veto. A well-known example is the permanent members of the United Nation Security Council, who have the right to veto any proposal with a single vote. A single-vote veto function was endowed to the majority logic gate with TBHQ as the input due to its strong inhibition of the colorimetric system. TBHQ can disable the entire system, resulting in a low absorption response, Fig. 5A(i). The majority logic gate will make a *FALSE* decision if TBHQ is introduced, implementing the single-vote veto function.

## Conclusions

Based on the catalytic activity of DNA combined with K^+^, Cu^2+^ and an antioxidant, three cascade logic gates including AND-OR-INH, AND-INH, and OR-INH were successfully constructed on a simple, universal and versatile molecular platform for the first time, which will augment the molecular computing power. By modulating the concentration of Cu^2+^, a majority logic gate with a single-vote veto function was conceptually mimicked on the same platform and triggered by the same set of inputs. The logic gates share a common threshold value and do not require an elaborate design or special synthesis, having the advantages of being enzyme-free, cost-effective, and simple to operate. Additionally, the logic system can act as a biosensor for high-sensitivity detection of antioxidant. This strategy is quite straightforward and provides an intriguing method for the fabrication of cascade logic gates and integration of multiple bioelectronics and biosensors on a single platform. Although the provided system has exciting prospects, the investigation is still in the experimental stage. There is a long way to go before we will have a ready-to-use molecular logic device.

## Methods

### Chemicals

Guanine-rich DNA oligonucleotide (DNA sequence: 5′-GTGGGTAGGGCGGGTTGG-3′, short named as G-DNA) was synthesized by Shanghai Sangon Biological Engineering Technology Co. Ltd. (Shanghai, China). 3,3′,5,5′-tetramethylbenzidine (TMB), KCl, Cu(NO_3_)_2_, tert-Butylhydroquinone (TBHQ), hemin, H_2_SO_4_, H_2_O_2_ (30% w/v) were purchased from Sigma-Aldrich (St. Louis, MO). The other chemicals were purchased from Aladin (Shanghai, China) and used as received without further purification. All other chemicals not mentioned here were of analytical reagent grade and used as received. Milli-Q ultrapure water (18.2 MΩ) was used throughout.

### Logic operation

DNA solutions were prepared by dissolving DNA in buffer (25 mM Tris-MES free of any metal ions, pH 8.0) and quantified by measuring UV absorption at 260 nm. Before use, the DNA solutions were heated at 90 °C for 10 min and gradually cooled to room temperature. To perform the logic functions, various combinations of inputs were added to the logic system, a mixture of TMB (0.25 mM), H_2_O_2_ (1.25 mM), hemin (200 nM) in buffer solution (20 mM Tris-MES, pH 5.0). DNA (400 nM), K^+^ (20 mM), Cu^2+^ (40 μM) and TBHQ (10 μM) were the inputs for cascade logic operations and DNA (400 nM), K^+^ (20 mM), Cu^2+^ (20 μM) and TBHQ (10 μM) were used for majority logic operations. After incubation for 90 min, the colorimetric reaction was stopped by adding an equal volume of 2 M H_2_SO_4_.

### Apparatus

The ultraviolet-visible (UV-Vis) spectra were recorded with a Cary 50-Bio UV spectrometer (Victoria, Australia). The CD spectra of the DNA (the concentration was 10 μM) in HEPES-MES buffer were collected by a Bio-Logic MOS450 (Bio-Logic, France). During experiments, the lamp was always kept under a stable stream of dry highly purified nitrogen. Three scans at 0.1 nm intervals were accumulated and averaged.

## Electronic supplementary material

Supplementary information
