# Supplementary material for: Implementation of cascade logic gates and majority logic gate on a simple and universal molecular platform
Source: Sci Rep. 2017 Oct 25;7:14014. doi: 10.1038/s41598-017-14416-7 (PMC5656625; doi:10.1038/s41598-017-14416-7)
Supplement: Supplementary file 1 — Supplementary information [file 41598_2017_14416_MOESM1_ESM.doc]

**Supporting Information**

**Implementation of cascade logic gates and majority logic gate on a simple and universal molecular platform**

Jinting Gao, Yaqing Liu,* Xiaodong Lin, Jiankang Deng, Jinjin Yin, Shuo Wang*

Key Laboratory of Food Nutrition and Safety (Ministry of Education of China), College of Food Engineering and Biotechnology, Tianjin University of Science and Technology, Tianjin Economic and Technological Development Area, the 13^th^ Avenue, No. 29, Tianjin 300457, China Corresponding authors: E-mail: yaqingliu@tust.edu.cn, s.wang@tust.edu.cn. Tel: (+86 22) 60912484, Fax: (+86 22) 60912489

**Results and discussion**


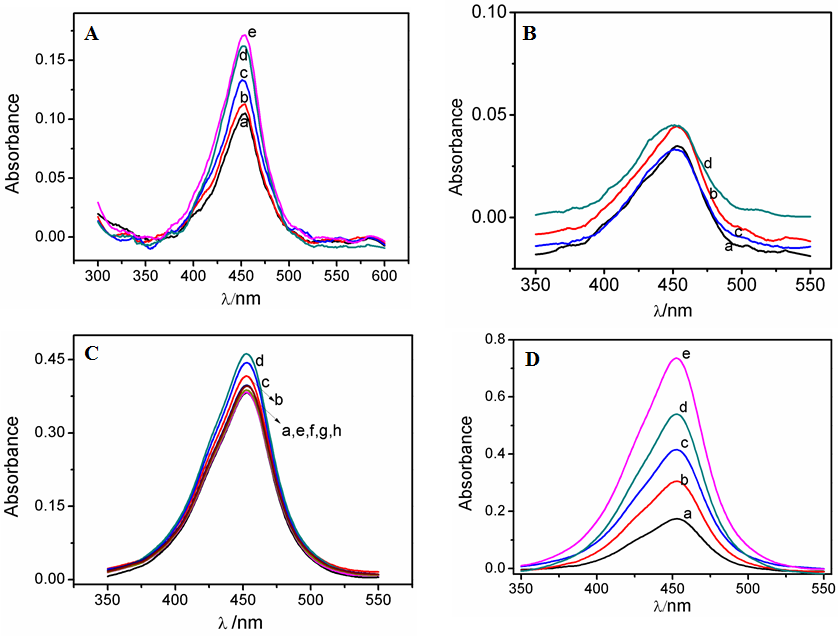


**Fig. S1.** UV-vis absorption spectra of the colorimetric system against A) G-DNA with different concentration: a) 50 nM, b) 100 nM, c) 200 nM, d) 400 nM, e) 500 nM; B) K^+^ with different concentration: a) 0 mM, b) 10 mM, c) 20 mM, d) 30 mM; C) coexistence of G-DNA (400 mM) and K^+^ with different concentration: a) 1 mM, b) 5 mM, c) 10 mM, d) 15 mM, e) 20 mM, f) 30 mM, g) 40 mM, h) 50 mM; D) coexistence of Cu^2+^ (20 uM) and K^+^ with different concentration: a) 0 mM, b) 10 mM, c) 20 mM, d) 30 mM, d) 50 mM.

The optimum experimental condition was explored by considering the requirements of majority logic gate and AND-OR-INH logic gate. As illustrated in Fig. S1, the colorimetric reaction of the system is not significantly influenced with increasing concentrations of G-DNA (Fig. R1A) and K^+^ (Fig. S1B). Cu^2+^ exhibits peroxidase-like activity on the colorimetric reaction of TMB system, which increases with increasing concentration Cu^2+^ (See Fig. 3A in the main text). In this case, 400 nM was first selected as DNA concentration according to our previous experience. Then, we investigated the colorimetric responses of the system in the coexistence of G-DNA (400 nM) and K^+^ with different concentration, Fig. S1C. The result indicates two points. One is that the colorimetric response of the system is significantly enhanced due to the formation of G4/Hemin complexes. The other is that the concentration of K^+^ within the explored range has no obvious influence on the colorimetric reaction. Here, it was noted that the absorption intensity reaches about 0.4, which can help to select the concentration of Cu^2+^ to fulfill the requirement of majority logic gate. A low output signal is read in the presence of each input for a three-input majority logic gate. Thus, 20 uM was selected as concentration of Cu^2+^ for the majority logic gate, which causes a lower output signal than the coexistence of G-DNA and K^+^. With the selected concentration of Cu^2+^, we further investigated the colorimetric response of the system in the coexistence of Cu^2+^ (20 uM) and K^+^ with different concentration, Fig. S1D. It was found that the coexistence of Cu^2+^ (20 uM) and K^+^ with concentration of 20 mM results in a similar absorption response as that in the coexistence of G-DNA (400 nM) and K^+^ (20 mM). Therefore, 20 mM was selected as concentration of K^+^. According to the above results, the function of cascade AND-OR-INH gate can be realized by just modulating concentration of Cu^2+^ as 40 uM.


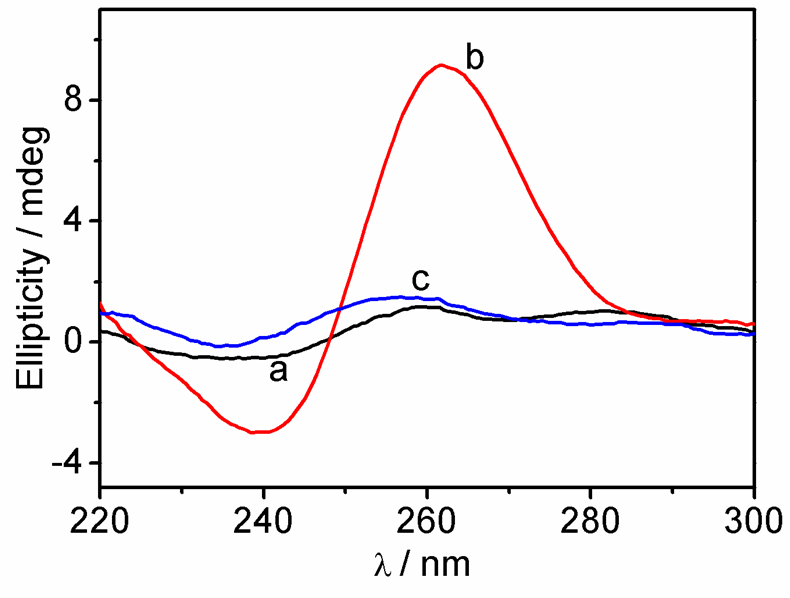


**Fig. S2.** Circular dichroism spectra of G-DNA (a) in the presence of K^+^ (b) and Cu^2+^ (c).

As illustrated in Figure S2, the circular dichroism (CD) spectrum of G-DNA is of relatively low amplitude, indicating a random strand structure, Figure 1B (a), which is significantly changed once K^+^ is added, Figure S2 (b). A negative peak near 240 nm and a positive peak near 262 nm are monitored, suggesting the forming of K^+^ stabilized G-quadruplex (G4) with a parallel configuration.^1,2^ No obvious configuration change of DNA is monitored from CD results if Cu^2+^ is added, Fig. S2 (c).


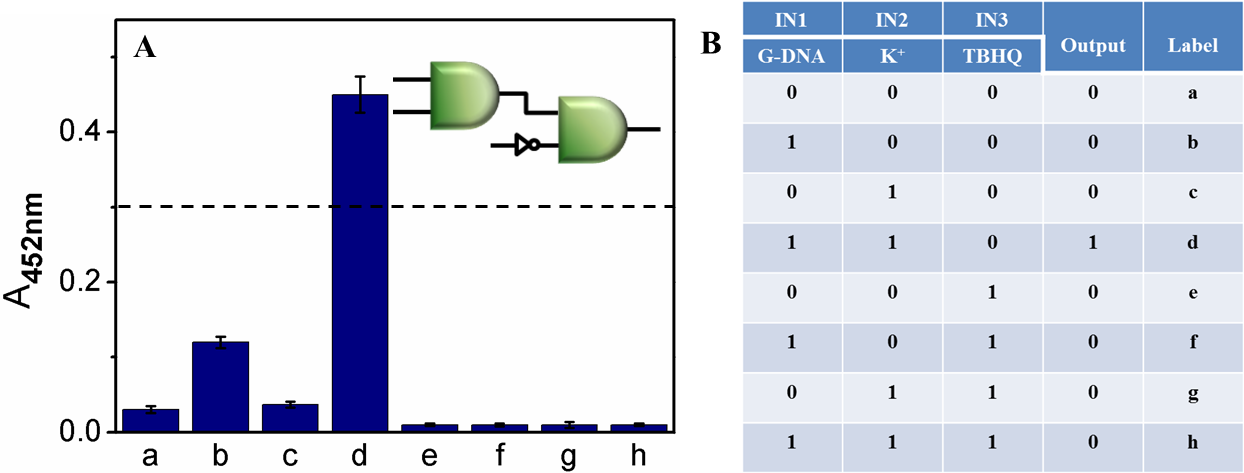


**Fig. S3.** Column bar of A_452nm_ against various input combinations with corresponding AND-INH logic circuit (A) and truth table (B). The error bar (mean ±SD) is obtained according to three independent experimental results.

According to the discussed results from Fig. 2 to Fig. 4 in the main text, AND-INH logic function can be performed by starting AND gate with G-DNA (IN1) and K^+^ (IN2) as the two inputs as discussed in Fig. 2. To perform the second-level INH gate, TBHQ is used as the third input (IN3) of the downstream gate which can disable colorimetric reaction of the entire system as shown in Fig. 3B in the main text. With the output of AND gate and TBHQ as inputs, an INH logic gate is then realized. By plotting A_452nm_ against various input combinations, a column bar with corresponding logic circuit and truth table are shown in Fig. S3, meeting the requirements of AND-INH logic operation.

**
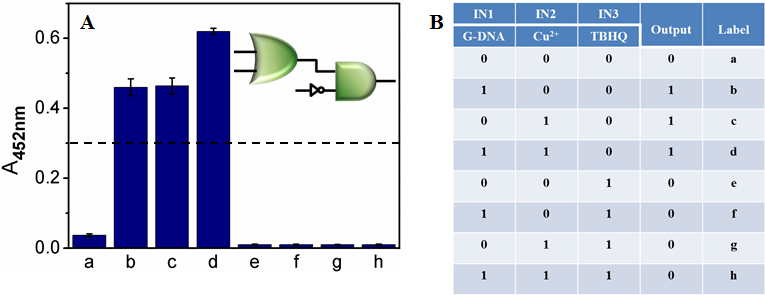
**

**Fig. S4.** Column bar of A_452nm_ against various input combinations with corresponding OR-INH logic circuit (A) and truth table (B). The error bar (mean ±SD) is obtained according to three independent experimental results.

To perform OR-INH logic function, K^+^ is first added into the platform, generating a low output as shown in Fig. 2A (c) in the main manuscript. OR logic gate is constructed with G-DNA (IN1) and Cu^2+^ (IN2) as the two inputs. Each of the input and the coexistence of the two inputs cause high output signal as demonstrated in Fig. 2A (d), Fig. 3B (b) and Fig. 3B (c), respectively. An OR gate is then realized. To implement INH gate function, TBHQ is introduced as the third input (IN3) by integrating the output of the OR logic gate. By plotting A_452nm_ against various input combinations, a column bar with corresponding logic circuit and truth table are shown in Figure S4, fulfilling the requirements of OR-INH logic operation.


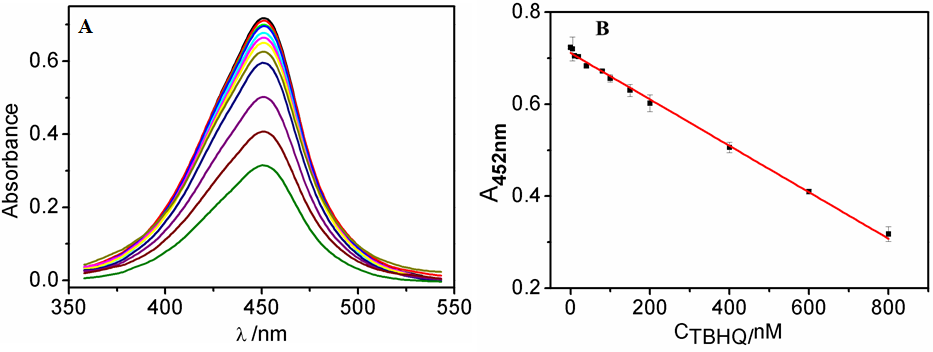


**Fig. S5.** UV-vis absorption spectra of TMB/H_2_O_2_/Hemin in the presence of G-DNA, K^+^ and Cu^2+^ as a function of concentration of TBHQ. The concentration of TBHQ is 0, 5, 10, 20, 40, 80, 100, 150, 200, 400, 600, 800 nM from up to bottom. Error bar (mean ±SD) is obtained on the basis of three independent experimental results.

The present system is expected not only to implement multiple logic functions, but also to supply detection information on antioxidant for food safety protection which is important for taking precautions against disease. TBHQ is one kind of synthetic phenolic antioxidants and is usually used to prevent pharmaceutical, food and other commercial products from oxidative rancidity.^3^ While, excess intake of antioxidants may result in a loss of nourishment and even generate toxic substances to harm people’s health.^4^ Considering that the synthetic antioxidants are hard to be decomposed and would accumulate in the body, it is of importance to limit the use of antioxidants on the point of keeping human health. Learned from Figure 3A (h), a significant absorption response of the system is monitored in the coexistence of G-DNA, K^+^ and Cu^2+^, which play function of signal amplification. After adding TBHQ, the absorption of TMB decreases with increasing concentration of TBHQ as illustrated in Fig. S5 (A). By plotting A_452nm_ against concentration of TBHQ, Fig. S4 (B), a detection limit of 5 nM with wide linear range from 5 nM to 800 nM is obtained, which is more sensitive than previous reports in Table S1^5,6^.

**Table S1** Comparison of different methods for TBHQ detection

| Detection methods | Linear range | Limit of detection | References |
| --- | --- | --- | --- |
| Electrochemical method | 10-50 μM | 75 nM | 7 |
| Electrochemical method | 1.05-10.10 μM | 34.3 nM | 8 |
| Electrochemical method | 0.20-2.80 g/mL | 0.079 g/mL | 9 |
| GC-MS | 0.100-20.0 mg/L | 11.5 μg/L | 10 |
| Colorimetric method | 5-800 nM | 5 nM | This work |


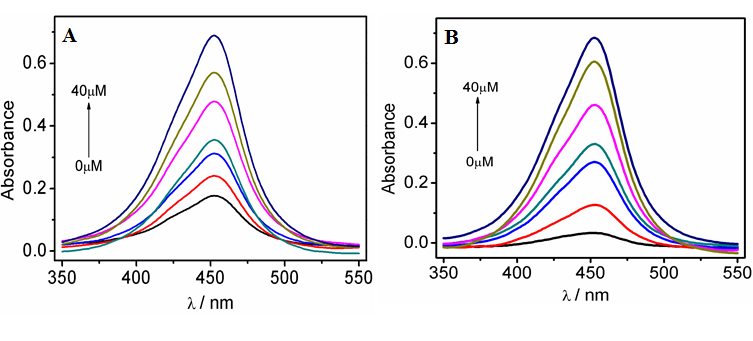


**Fig. S6.** UV-vis absorption spectra of TMB/H_2_O_2_/Hemin system in the presence of G-DNA (A) or K^+^ (B) against concentration of Cu^2+^: 0, 1, 5, 10, 20, 30, 40 μM from bottom to up.

As illustrated in Fig. S6, the absorption responses of the TMB increase with increasing concentration of Cu^2+^ in the presence of G-DNA (A) or K^+^ (B).

**References**:

1. Majhi, P. R. & Shafer, R. H. Characterization of an unusual folding pattern in a catalytically active guanine quadruplex structure*. Biopolymers* **82**, 558-569 (2006).

2. Liu, Y. Q. *et al*. An aptamer-based keypad lock system. *Chem. Commun.* **48**, 802-804 (2012).

3. Guo, Y. Q., Chu, Q. C., Fu, L., Wu, T. & Ye, J. N. Determination of phenolic antioxidants by micellar electrokinetic capillary chromatography with electrochemical detection. *Food Chem*. **94**, 157-162 (2006).

4. Chung, J. G. Effects of butylated hydroxyanisole (BHA) and butylated hydroxytoluene (BHT) on the acetylation of 2-aminofluorene and DNA-2-aminofluorene adducts in the rat. *Toxicol. Sci.* **51**, 202-210 (1999).

5. Lin, X. Y., Ni, Y. N. & Kokot, S. Glassy carbon electrodes modified with gold nanoparticles for the simultaneous determination of three food antioxidants. *Anal. Chim. Acta* **765**, 54-62 (2013).

6. Guo, L., Xie, M. Y., Yan, A. P., Wan, Y. Q. & Wu, Y. M. Simultaneous determination of five synthetic antioxidants in edible vegetable oil by GC–MS. *Anal. Bioanal. Chem.* **386**, 1881-1887 (2006).

7. Tormin, T. F., Cunha, R. R., Richter, E. M. & Munoz, R. A. A. Fast simultaneous determination of BHA and TBHQ antioxidants in biodiesel by batch injection analysis using pulsed-amperometric detection. *Talanta* **99**, 527-531 (2012).

8. De Araujo, T. A., J. Barbosa, A. M., Viana, L. H. & Ferreira, V. S. Electroanalytical determination of TBHQ, a synthetic antioxidant, in soybean biodiesel samples. *Fuel* **90**, 707-712 (2011).

9. Lin, X. Y., Ni, Y. N. & Kokot, S. Glassy carbon electrodes modified with gold nanoparticles for the simultaneous determination of three food antioxidants. *Anal. Chim. Acta.* **765**, 54-62 (2013).

10. Guo, L., Xie, M. Y., Yan, A. P. & Wu, Y. M. Simultaneous determination of five synthetic antioxidants in edible vegetable oil by GC–MS. *Anal. Bioanal. Chem.* **386***,* 1881-1887 (2006).
